# Quality of care in the ICU from the perspective of relatives

**DOI:** 10.1186/cc12481

**Published:** 2013-03-19

**Authors:** M Van Mol, E Bakker, A Rensen, I Menheere, L Verharen

**Affiliations:** 1 Erasmus MC, Rotterdam, the Netherlands; 2 OU, Heerlen, the Netherlands; 3 HAN, Nijmegen, the Netherlands

## Introduction

This study describes the development and validation of the Consumer Quality Index Relatives in ICUs (CQI 'R-ICU'), which aims to measure the satisfaction of relatives and to identify aspect of care that need improvement in the ICU in a reliable and valid way. According to the quality standards of the Dutch Society of Intensive Care, every ICU needs to record the satisfaction of relatives [1]. At this moment there is insufficient insight into the quality of care offered to relatives on the ICU because an evidence-based Dutch measurement instrument is missing.

## Methods

The CQI 'R-ICU' has been developed based on a scientific and standardised method [2]. A mixed design method is used, consisting of qualitative and quantitative survey studies. Factor analyses are carried out to determine the underlying structure of the newly developed questionnaire. Multiple regression analysis is used to explore the relationship between demographic variables and the perceived quality of care.

## Results

In six hospitals the CQI 'R-ICU' is sent to relatives after receiving informed consent (*n *= 441), 55.1% of the respondents are the patient's partner. Respondents seem to be most satisfied with the presence of a professional at first entrance to the ICU. The highest need for improvement scores relate to information about meals, parking and other disciplines (for example, social worker, spiritual worker or psychologist). Factor analysis shows that quality of care is determined by four clusters of items: Support, Communication, General Information and Organisation. The reliability of the CQI 'R-ICU' is sufficiently high, only Communication and Support are significant predictors of total quality judgement of relatives (adj. *R*^2 ^= 0.74). In addition, there is a significant difference in mean total quality judgement between the six hospitals as well as between the four wards within Erasmus MC. None of the demographic variables such as sex, age, education, race and length of stay had an effect on perceived quality of care.

## Conclusion

The CQI 'R-ICU' turned out to be a valid, reliable, sensitive and feasible instrument. Large-scale implementation is recommended.

## References

[B1] Vos Quality measurement at intensive care units: which indicators should we use? J Crit Care 2007 22 267 274 10.1016/j.jcrc.2007.01.002 18086396

[B2] Sixma Handboek CQI Ontwikkeling: richtlijnen en voorschriften voor de ontwikkeling van een CQI meetinstrument 2008 Utrecht: Nivel

